# Doping concentration dependence of microstructure and magnetic behaviours in Co-doped TiO_2_ nanorods

**DOI:** 10.1186/1556-276X-9-673

**Published:** 2014-12-12

**Authors:** Li-Ting Tseng, Xi Luo, Thiam Teck Tan, Sean Li, Jiabao Yi

**Affiliations:** 1 School of Materials Science and Engineering, University of New South Wales, Sydney, NSW 2052, Australia

**Keywords:** TiO_2_, Nanorods, Ferromagnetism, Ti vacancy

## Abstract

Co-doped titanium dioxide (TiO_2_) nanorods with different doping concentrations were fabricated by a molten salt method. It is found that the morphology of TiO_2_ changes from nanorods to nanoparticles with increasing doping concentration. The mechanism for the structure and phase evolution is investigated in detail. Undoped TiO_2_ nanorods show strong ferromagnetism at room temperature, whereas incorporating of Co deteriorates the ferromagnetic ordering. X-ray photoelectron spectroscopy (XPS) and electron spin resonance (ESR) results demonstrate that the ferromagnetism is associated with Ti vacancy.

## Background

Titanium dioxide (TiO_2_) is one of the popular wide gap semiconductors that have attracted much attention for decades. It has three main different polymorphs: anatase, rutile and brookite, which lead to different physical and chemical properties affecting the performance of its applications. Anatase TiO_2_ has been reported as a stable phase in photocatalyst applications [1]. Rutile TiO_2_ is a high temperature stable phase with a bandgap of approximately 3.0 eV, which is slightly smaller than the anatase phase (approximately 3.2 eV). Whereas, brookite phase is less stable compared to the other two crystal structures and can only be prepared under specific conditions [2]. TiO_2_ has been extensively studied because of the variety in crystal structures and properties giving rise to its promising applications including photocatalyst, lithium electrode, sensors, solar cells and data storage technology as well as spintronic devices [3-8]. Nanostructured TiO_2_ is one of the most important categories in TiO_2_ for practical applications in photovoltaic, photocatalyst and supercapacitors, as well as batteries [9]. Nanostructured TiO_2_ has been synthesized by many techniques, including hydrothermal method [10] and chemical vapour deposition method [11,12].

In order to modify the properties to meet the requirements for certain applications, TiO_2_ is usually doped with a variety of elements, such as light elements (S, F or N), transition metals (Co, Ni, Fe) or rare earth elements (Nd, Eu). It has been well known that doping with light element or rare earth element can eminently narrow the bandgap, which will enhance photocatalyst and solar absorption properties [13,14]. The doping using transition metals may produce a new material, called diluted magnetic semiconductor (DMS), which combines both magnetic and semiconducting behaviour. This material is promising for spintronic device applications, which can provide devices with high density, high capacity, high speed and multifunction using the dissipationless spin current.

Co-doped TiO_2_ is the first oxide-based DMS that shows room temperature ferromagnetism [15]. It has attracted wide interest due to its promising properties for spintronic devices. Most of the researches have focused on Co-doped TiO_2_ thin films, which were commonly prepared by pulsed laser deposition (PLD) and molecular beam epitaxy (MBE). Nanostructured diluted magnetic semiconductors have been paid much attention due to their unique properties, such as giant Zeeman splitting and tuneable magnetic properties [16,17]. However, most of these investigations focus on nanoparticles [18] and only few studies worked on TiO_2_ nanowires or nanorods [19-21]. It may be due to the difficulty for the control of shapes and sizes of TiO_2_ nanowires or nanorods during the synthesis process as well as the low production yield. Although introducing dopants into TiO_2_ in the nanostructured state is still a challenge due to the self-purification effects, efforts have been made to fabricate TiO_2_ nanorods/nanowires for its importance of the realization of microelectronic devices. Recently, Liu et al. synthesized transition-metal-doped TiO_2_ nanowires with large quantities and high quality using molten salt synthesis [22]. Molten salt synthesis is one of the promising methods to prepare ceramic powders, which is able to control the sample morphology and size. In addition, this method has the advantage to enhance the reaction of powder precursors [23]. They demonstrated the successful incorporation of transition metal ions into the TiO_2_ lattice. It has been proven to be a very simple and economical method that can provide an effective way for fabricating high quality TiO_2_ based DMS nanostructures.

In this work, rutile TiO_2_ nanorods doped with various cobalt concentrations were synthesized using a molten salt method. The effects of doping concentration of Co on the microstructure and magnetic properties of Co-doped TiO_2_ DMS, as well as the formation mechanism of TiO_2_ nanorods, were studied. The results revealed that Co concentration strongly affected the morphology of the nanostructures. TiO_2_ nanorods were successfully grown under low Co-doping concentrations. As Co doping increases, a small amount of cobalt oxide might precipitate, which is coated onto the surface of TiO_2_ nanostructures to restrain the formation of nanorods, resulting in the formation of nanoparticles. In addition, the incorporation of Co strongly suppresses the ferromagnetic ordering, suggesting that the ferromagnetic ordering may not originate from Co doping. Detailed investigations indicate that the complex defects related to Ti vacancy are attributed to the ferromagnetism in the undoped TiO_2_ nanostructures. The incorporation of Co during fabrication impedes the formation of complex defects, leading to the deterioration of ferromagnetic ordering.

## Methods

Rutile TiO_2_ nanorods were synthesized using a molten salt method. The powders of TiO_2_ (Aeroxide® P25, mixed phases of anatase and rutile with a proportion of 80:20), Na_2_HPO_4_ and NaCl with an impurity higher than 99% were mixed with a ratio of 1:1:4. The precursors were then ground with an alumina mortar. All the chemicals were purchased from Sigma-Aldrich, Castle Hill, NSW, Australia. The mixtures of the powders was placed into a crucible and heated to 1,098 K in a muffle furnace for 8 h. The product powders were washed with deionized water to remove NaCl and other impurities by a centrifuge (Beckman Coulter Allegra X-30 Centrifuge (Beckman Coulter, Fullerton, CA, USA)). Then, the powder precipitates were washed by ethanol and placed steadily in the oven at 353 to 363 K for drying. Co_3_O_4_ powder was added into the mixture to make Co-doped TiO_2_ nanorods with preset Co-doping concentrations of 0.5, 1, 3, 5 and 10 at%. Post-annealing was carried out at 773 K for 30 min under Ar atmosphere for all samples.

The crystal structures of powder samples were analysed by an X-ray diffractometery (XRD, PANalytical Xpert Multipurpose X-ray Diffraction System (PANalytical, Almelo, The Netherlands), Cu Kα radiation). The morphology and structures of samples were examined by scanning electron microscopy (SEM, FEI Nova NanoSEM 230, Hillsboro, Oregon, USA) and transmission electron microscopy (TEM, Philips CM200, Hillsboro, Oregon, USA). Electronic structure and chemical compositions were characterized by X-ray photoelectron spectroscopy (XPS, ThermoScientific ESCALAB 250i X-ray Photoelectron Spectrometer (ThermoScientific, Waltham, MA, USA)) and inductively coupled plasma mass spectrometry (ICPMS, PerkinElmer quadrupole NexION ICP-MS (PerkinElmer, Melbourne, Victoria, Australia)), respectively. Superconducting quantum interference device (SQUID, Quantum design-XL-7, San Diego, CA, USA) was used for measuring magnetic hysteresis (M-H) loops with a maximum magnetic field of 2 Tesla at room temperature. Electron spin resonance (ESR) spectrum (Bruker EMX-plus X-Band ESR Spectrometer (Bruker, Karlsruhe, Germany)) was taken for studying the magnetic behaviours.

## Results and discussion

**Figure 1:**
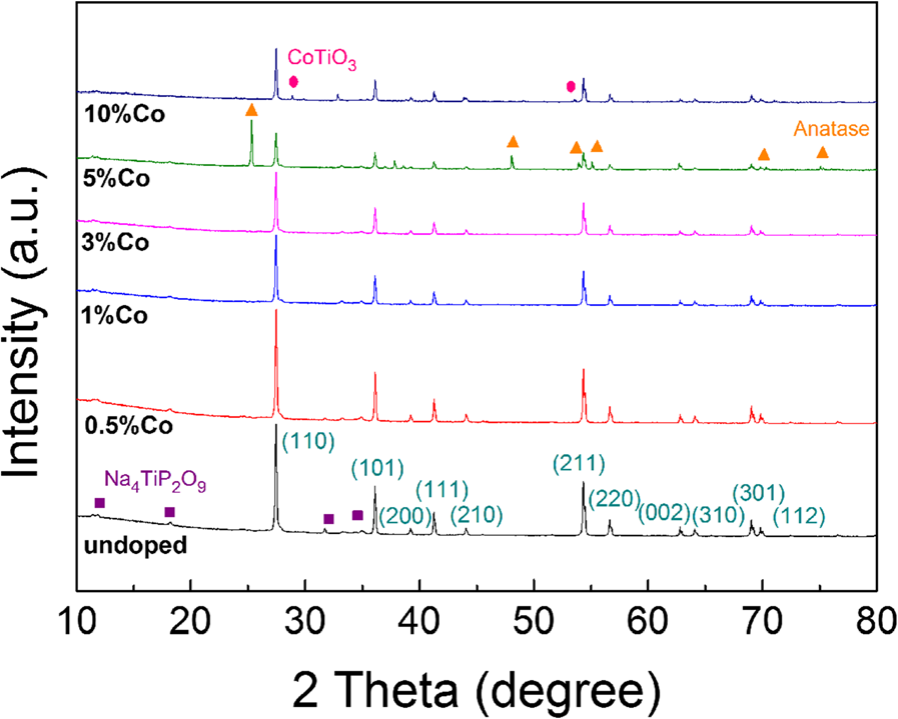
**XRD spectra of TiO**_**2 **_**nanorods with various Co concentrations.** Pink circle: CoTiO_3_; orange triangle: anatase TiO_2_; and violet square: Na_4_TiP_2_O_9_.

Figure 1 shows XRD results of undoped and Co-doped TiO_2_ nanorods with different doping concentrations. It can be seen that only the rutile phase is identified in TiO_2_ nanorods prepared with and without Co doping up to 3 at%. As the Co doping is increased to 5 at%, both the anatase and rutile phases were detected. Further increasing the Co doping to 10 at%, a secondary phase of CoTiO_3_ is observed. It is also noted that monoclinic tetrasodium titanium nonaoxodiphosphate (Na_4_TiP_2_O_9_) is found in all the samples, which is an intermediate phase during the reaction process to facilitate the growth of rutile nanorods [22].

**Figure 2:**
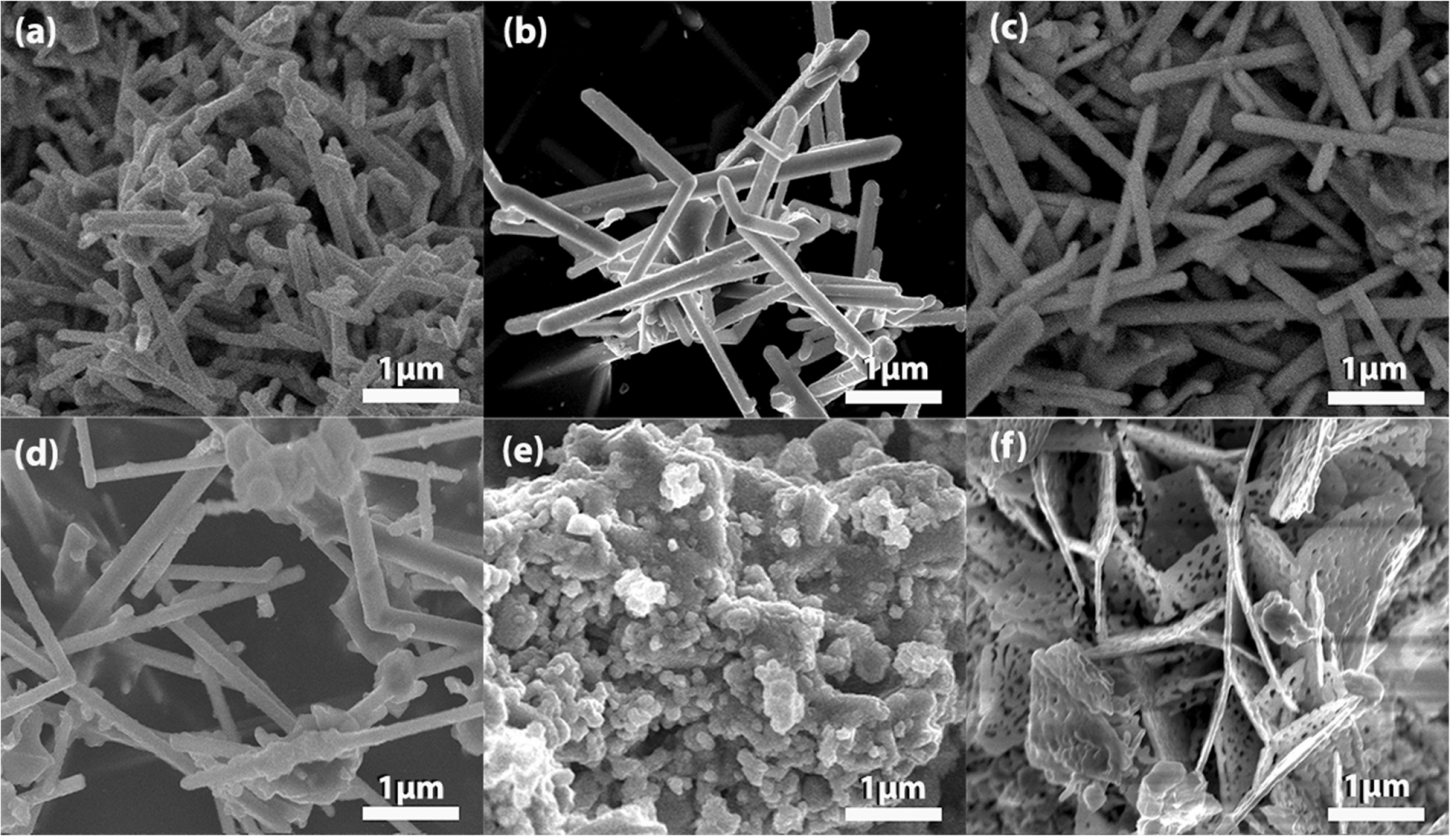
**SEM images of TiO**_**2 **_**samples with different Co concentrations. (a)** Undoped, **(b)** 0.5, **(c)** 1, **(d)** 3, **(e)** 5 and **(f)** 10 at% Co.

Figure 2 shows the SEM images of TiO_2_ nanorods with and without Co doping, indicating that TiO_2_ nanorods have been successfully synthesized. It appears that the undoped sample is relatively short in length compared with that of doped samples as shown in Figure 2a,b,c,d. This suggests that a small amount of Co doping facilitates TiO_2_ nanorods growing along the [001] direction. The diameter and length of the nanorods are in the range of 100 to 200 nm and 1 to 3 μm, respectively. It can be seen from the images that the nanorods are thickened and nanoparticles are observed when the doping concentration of Co is increased to 3 at%. When the doping concentration of Co increases to 5 at%, the morphology changes from nanorods to nanoparticles, as seen in Figure 2e. Further increasing the doping concentration to 10 at%, the nanoparticles change to nanoflakes with mesoporous pores (Figure 2f). From these results, it can be concluded that Co concentration plays an important role in the growth process that affects both the phase transformation and particle shapes.

**Figure 3:**
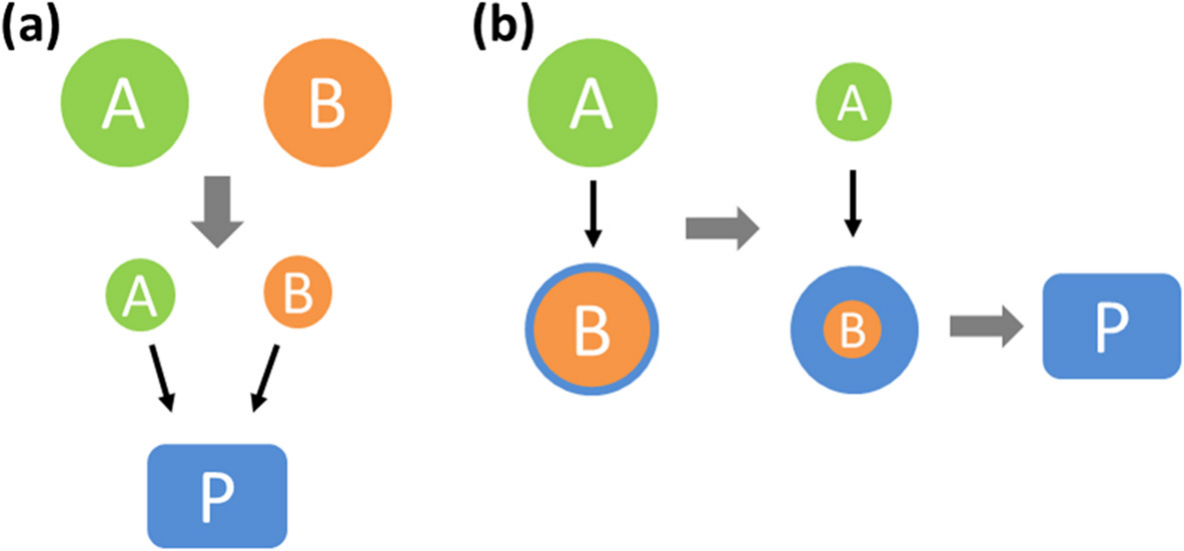
**Growth mechanism of molten salt synthesis. (a)** Solution-precipitation process and **(b)** solution-diffusion process (modified from [23]).

In the molten salt process, the formation of the nanostructures is highly related to relative dissolution rate of reactants [23]. If the dissolution rates of two reactants (i.e. TiO_2_ and Na_4_TiP_2_O_9_) are similar, both reactants would dissolve in the molten salt with the same rate and form the nanostructures once it reaches supersaturated condition. In this case, the nanostructured precipitation is based on homogeneous nucleation (Figure 3a). However, in another scenario (Figure 3b), if the dissolution rate of one reactant is higher than the other, the reactant with a higher dissolution rate would be dissolved into the molten salt first and then the dissolved reactant would coat on the reactant with a lower dissolution rate, as shown in situation B, rather than diffuse homogeneously into the reactant. Such a processing produces a core-shell structure. Then, the diffusion between two reactants takes place to form a product layer on the surface of the reactant as long as a sufficient amount of activation energy is provided. In contrary to situation A, the final product may not undergo the whole diffusion process, which affects its morphologies and compositions.

**Figure 4:**
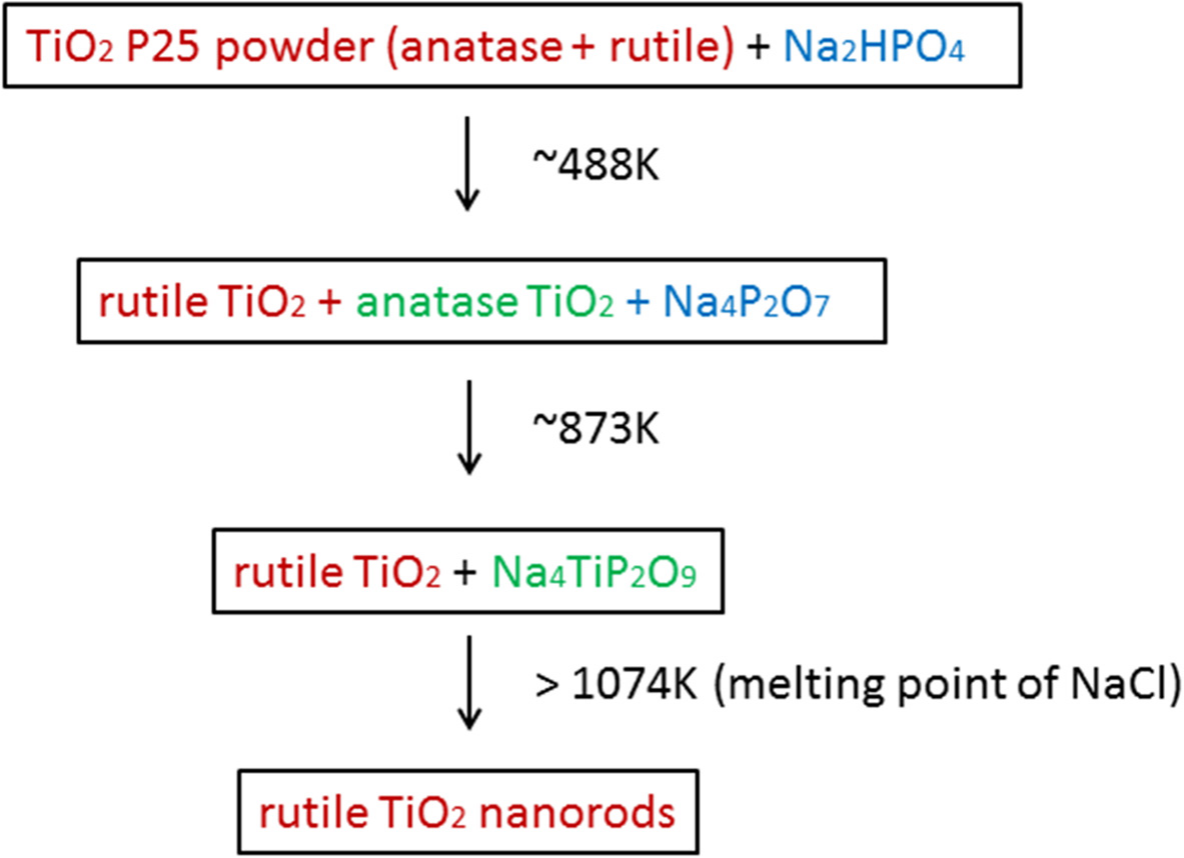
Reactions flow chart of molten salt synthesis.

In this work, the growth of TiO_2_ nanorods is based on scenario B due to the different dissolution rate of TiO_2_ and Na_4_TiP_2_O_9_. P25 powders with particle sizes about 21 nm were used in our experiments, which contain both anatase and rutile phases. During the synthesis process, dibasic sodium phosphate (DSP, Na_2_HPO_4_) decomposed to Na_4_P_2_O_7_ + H_2_O at approximately 473 K [24]. The compound of Na_4_P_2_O_7_ would react with anatase TiO_2_ (the metastable form of TiO_2_) to form an intermediate phase of Na_4_TiP_2_O_9_ at 873 K that promotes the growth of rutile TiO_2_ nanorods. The rutile phase in the precursors acts as the seeds to facilitate the heterogeneous nucleation and growth process [22]. Part of anatase TiO_2_ particles may undergo phase transformation to the rutile TiO_2_ at high temperature. Rutile TiO_2_ particles do not react with Na_2_HPO_4_ as both of them are thermodynamically stable at high temperature. Once the temperature reaches the melting point of salt (1,074 K), rutile TiO_2_ and Na_4_TiP_2_O_9_ will dissolve into the salt solution. When the supersaturate state of the dissolved reactants is reached, precipitation occurs on rutile TiO_2_ particles. The reactant with a higher dissolution rate, namely Na_4_TiP_2_O_9_, diffuses continuously through the molten salt and reaches the surface of TiO_2_ precipitates to facilitate the growth process [23]. The rutile TiO_2_ precipitates start to grow along the direction of [001] with the lowest solid-liquid interfacial energy [25,26]. Finally, rutile TiO_2_ nanorods are obtained when both the reactants are completely consumed. A flow chart represents the reaction steps as sketched in Figure 4.

For the sample doped with 5 at% Co, both anatase and rutile phases are observed and Na_4_TiP_2_O_9_ is also detected, which implies that anatase TiO_2_ in P25 precursor may undergo both the reaction with Na_4_P_2_O_7_ and the phase transformation to rutile phase. It is well known that the impurity may significantly affect the phase transformation of TiO_2_[27]. As the Co doping increases to 5 at%, the high doping concentration of dopants may inhibit the transformation of the anatase TiO_2_ into rutile phase [1]. On the other hand, Co atoms may form cobalt oxide (CoO) that is coated onto the surface of TiO_2_ and Na_4_P_2_O_7_ precipitates, thus suppressing the reaction between the anatase TiO_2_ and Na_4_P_2_O_7_. Consequently, the growth of nanorods is prohibited and nanoparticles are formed instead. When the Co doping reaches 10 at%, the relatively high Co content leads to the formation of CoTiO_3_ secondary phase, as detected by XRD. The morphology of TiO_2_ then changes from nanoparticles to nanoflakes (Figure 2f). The exact mechanism for the formation of nanoflakes is not clear. It may be related to the excess amount of Co and the formation of CoTiO_3_ in the sample. It had been reported that the impurity phases may affect the shapes of nanostructures [1,28].

In order to further understand the formation mechanisms of TiO_2_ nanorods, the experiments with varied synthesis conditions, such as calcination temperatures (1,098 and 1,173 K with 8-h duration), heating duration (2, 8 and 16 h at 1,098 K) and NaCl/DSP ratio (90/10, 80/20 and 60/40 at 1,098 K for 8 h) were carried out. The results are summarized in Table 1. From the table, it can be seen that the samples doped with 1 at% Co are all grown to nanorods. It demonstrated that the heating duration and NaCl/DSP ratio have very limited influence on the shapes and sizes of nanorods. However, when the heating duration of 5 at% Co-doped sample is increased to 16 h, only the rutile phase exists and a strong peak of Co_2_P_4_O_12_ is detected by XRD, indicating that the Co dopants reacted with Na_4_P_2_O_7_ to form Co_2_P_4_O_12_ secondary phase, which suppressed the formation of Na_4_TiP_2_O_9_.

**Table 1:** Summary of sample morphologies

	**Phase**	**Morphology**	**Secondary phase**
1,098 K/8 h			
Undoped	Rutile	Nanorod	-
0.5% Co	Rutile	Nanorod	-
1% Co	Rutile	Nanorod	-
3% Co	Rutile	Nanorod	-
5% Co	Rutile + anatase	Nanoparticle	-
10% Co	Rutile	Nanoparticle + nanorod	CoTiO_3_ (nanoflakes)
1% Co-doped			
1,098 K/8 h	Rutile	Nanorod	-
1,173 K/8 h	Rutile	Nanorod	-
2 h/1,098 K	Rutile	Nanorod	-
8 h/1,098 K	Rutile	Nanorod	-
16 h/1,098 K	Rutile	Nanorod	-
5% Co-doped			
1,098 K/8 h	Rutile + anatase	Nanoparticle	-
1,173 K/8 h	Rutile	Nanoparticle	-
2 h/1,098 K	Rutile + anatase	Nanoparticle	-
8 h/1,098 K	Rutile + anatase	Nanoparticle	-
16 h/1,098 K	Rutile	Nanoparticle	Co_2_P_4_O_12_

When the calcination temperature was raised to 1,173 K, the length of nanorods becomes longer than the sample calcinated at 1,098 K. Higher temperature gives rise to a higher solubility and diffusivity of reactants, which may accelerate the reaction rate, resulting in a larger dimension of nanorods. Moreover, it is noted that the anatase phase disappears when the calcination temperature reached 1,173 K. This may attribute to the higher activation energy at higher temperature, thus facilitating the phase transformation. For 5 at% Co-doped TiO_2_ sample, no nanorods were observed by varying the synthesis parameters and the nanoparticles are the often-observed products.

**Figure 5:**
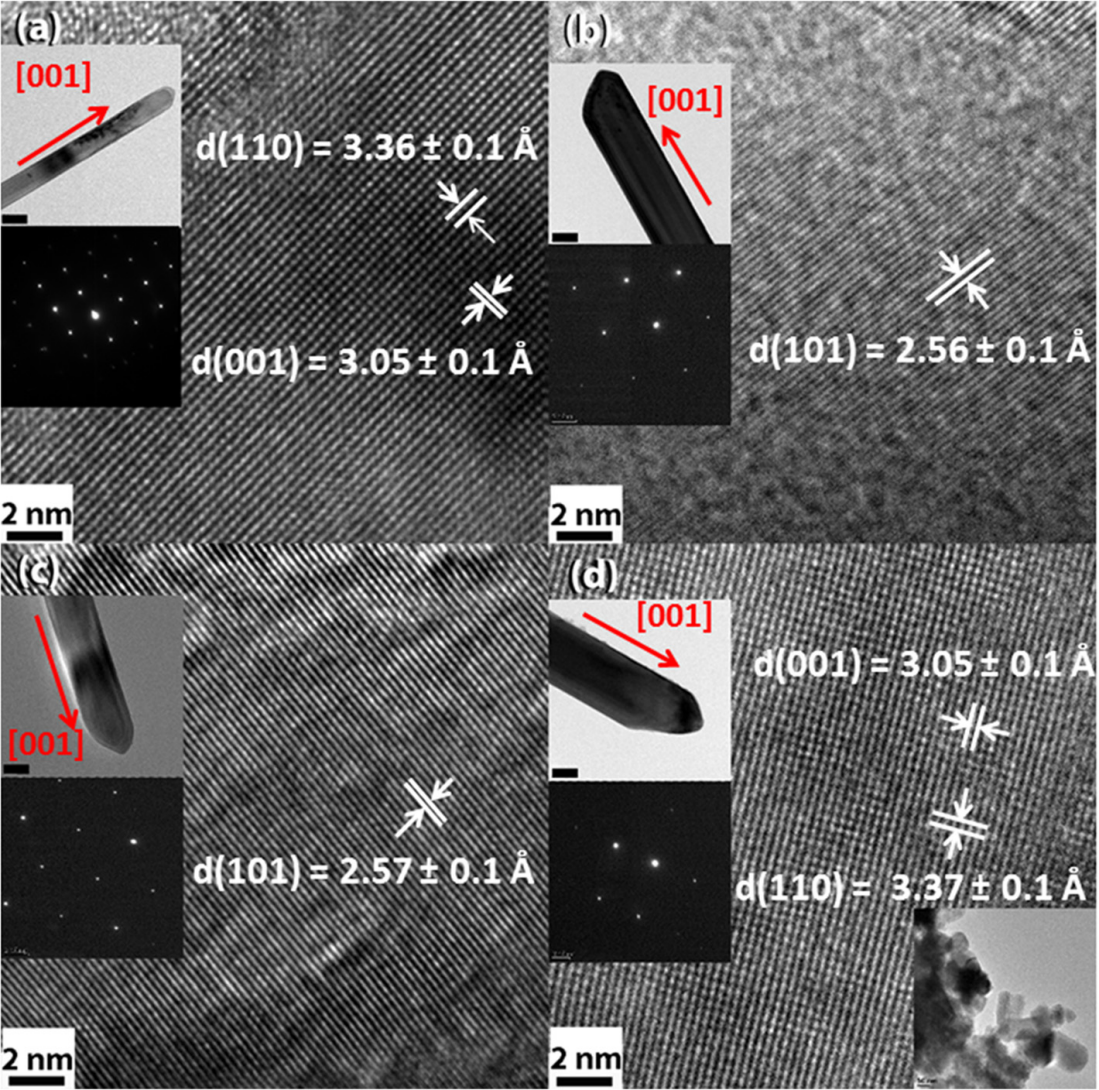
**High resolution TEM images of TiO**_**2 **_**nanorods. (a)** undoped, **(b)** 0.5, **(c)** 1 and **(d)** 3 at% Co. The insets in the images are nanorods at low magnification (scale bar = 100 nm) and SAED patterns. The inset in (d) at the right bottom is the TEM image of 5 at% Co-doped TiO_2_ nanoparticles.

Figure 5 shows TEM micrographs of TiO_2_ nanorods with different doping concentrations. All the samples grow along [001] direction, as shown in Figure 5a,b,c,d. High resolution TEM images and the selected area electron diffraction (SAED) patterns shown in the insets reveal the single crystal structure of the nanorods, indicating that there is no significant influence of Co doping on the crystallization processes.

The d spacings of lattice planes of the rutile TiO_2_ nanorod are measured to be approximately d_001_ = 3.05 Å, d_110_ = 3.37 Å and d_101_ = 2.55 Å, which agrees well with the reported results [22]. The nanorods were grown along the *c*-axis with the lowest solid-liquid interfacial free energy that was not affected by the presence of Co. This result is consistent with that reported by Liu et al. [22]. The inset in the bottom of Figure 5d shows the morphologies of nanoparticles for 5 at% Co-doped TiO_2_, which is in consistence with that observed by SEM.

**Figure 6:**
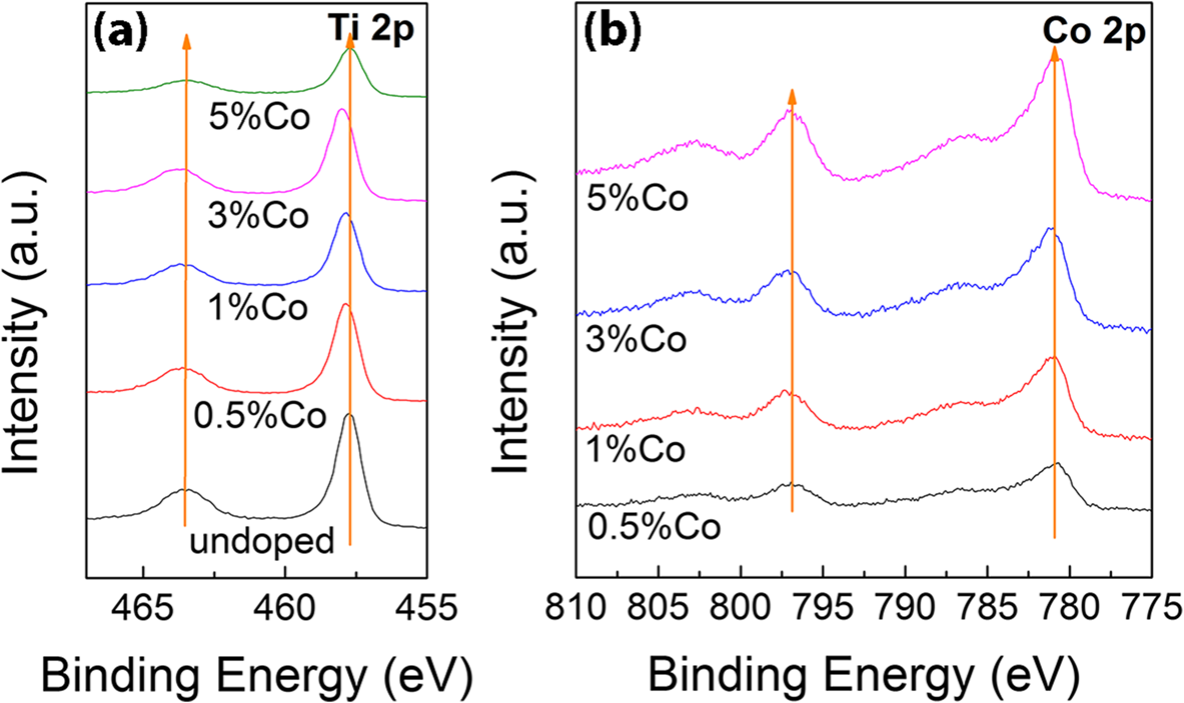
**XPS spectra of TiO**_**2 **_**nanorods with various Co concentrations. (a)** Ti 2p and **(b)** Co 2p.

For the composition of the nanostructures, inductively coupled plasma (ICP) analysis indicates that the Co-doping concentration is very close to the nominal concentration. However, chemical composition analysed by XPS shows a much higher Co concentration than that obtained by ICP. These results imply that Co may mainly segregate on the surface of TiO_2_ nanorods due to the nature of the surface sensitivity of XPS. XPS spectra of Ti 2p edge are shown in Figure 6a. Ti 2p_3/2_ and Ti 2p_1/2_ peaks are detected at 457.74 to 458.98 eV and 463.37 to 463.71 eV, respectively, which belongs to Ti^4+^. The separation of Ti 2p peaks (ΔTi 2p) is a characteristic to identify the crystal structure of TiO_2_. A ΔTi 2p value of 5.77 eV represents Ti-O bonding in rutile TiO_2_[29,30], which is corresponding to the spectra of undoped and 1% Co-doped samples. Peak shifts can be observed in the samples doped with 3 and 5 at% Co, where ΔTi 2p decreases to 5.73 and 5.66 eV, respectively. The peak shift implies the existence of the anatase phase in the samples. Therefore, there might be a small amount of anatase TiO_2_ present in 3 at% Co-doped sample that cannot be detected by XRD. Two main peaks of Co 2p_3/2_ and Co 2p_1/2_ are observed at 781.01 to 781.24 eV and 796.97 to 797.33 eV, respectively, in Co 2p spectra (Figure 6b), followed by two satellite peaks at approximately 786 eV and 803 eV, respectively. The separation of Co 2p_3/2_ and Co 2p_1/2_ indicates that the valence state of Co is 2+. The spin-orbit splitting in our samples is approximately 16 eV, which is inconsistent with that reported in [31-34]. O 1 s is not shown here because of the presence of multioxide compounds in the samples.

**Figure 7:**
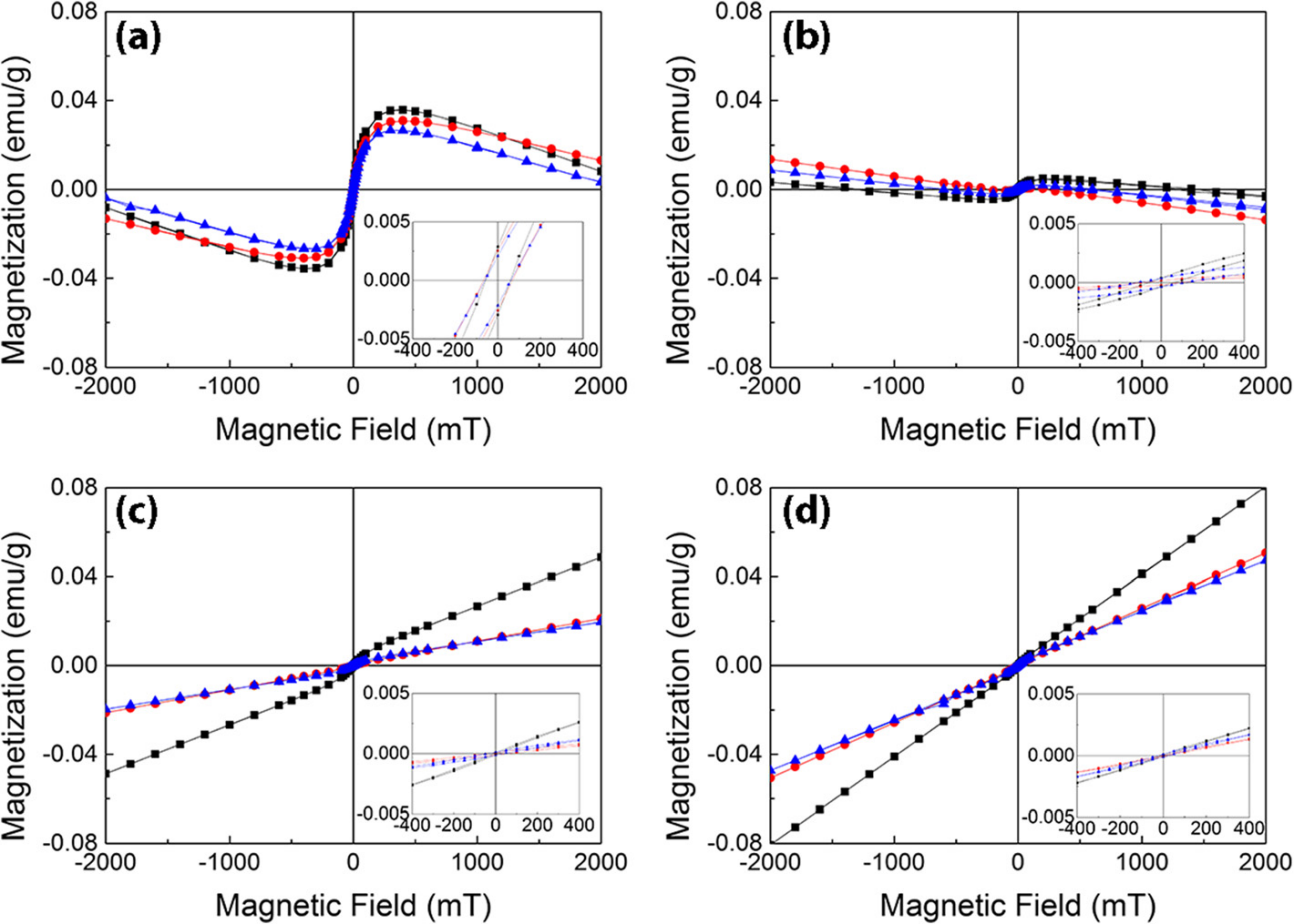
**Room temperature hysteresis loop of TiO**_**2 **_**nanorods with various Co concentrations. (a)** Undoped, **(b)** 0.5, **(c)** 1 and **(d)** 3 at% Co (black square: before annealing; red circle: after Ar annealing; and blue triangle: after O_2_ annealing).

Figure 7 shows room temperature M-H loops of TiO_2_ nanorods doped with different Co concentrations before and after annealing. The magnetic properties of the samples doped with 5 and 10 at% Co are not shown here because of the complexity of polymorphic TiO_2_ phases and the presence of CoTiO_3_ secondary phase. For the as-prepared nanorods, relative strong ferromagnetism can be observed in undoped and 0.5 at% Co-doped nanorods. In addition, diamagnetism can also be seen, suggesting not fully magnetic phases in the samples, which may be associated with the aforementioned residual reactants and reaction intermediates, i.e. Na_4_TiP_2_O_9_. For 1 and 3 at% Co-doped TiO_2_, the long-range ferromagnetic ordering no longer exists but antiferromagnetism or paramagnetism takes place.

The insets in Figure 7 show the magnification of the M-H curves in the central area. The coercivity shown for the undoped and 0.5 at% Co-doped TiO_2_ nanorods confirms the ferromagnetic ordering. Whereas, there is no coercivity observed in 1 and 3 at% Co-doped TiO_2_ samples, suggesting that Co doping may deteriorate long-range magnetic ordering. The XPS results suggest that Co ions do not incorporate into the TiO_2_ lattice but coat on the surface of the samples in the form of CoO, which could be in amorphous phase or with the small amount that cannot be detected by XRD. It is well known that CoO is an antiferromagnetic material [35]. In Co-doped TiO_2_ sample, surface segregation of CoO on TiO_2_ nanorods shows paramagnetism or antiferromagnetism, which leads to the decrease in ferromagnetism.

It is interesting to note that the undoped TiO_2_ nanorods have the strongest ferromagnetism among all the samples, suggesting that Co doping is not the origin of ferromagnetism. The origin of weak ferromagnetism observed in the undoped TiO_2_ has been widely discovered and could be owing to the presence of intrinsic point defects, such as oxygen vacancy [36], Ti vacancy [37-40] or Ti^3+^[41]. However, Ti^3+^ is not detected in XPS Ti 2p spectra (Figure 6a) and is more likely to form antiferromagnetic coupling [42]. Therefore, the ferromagnetism may possibly come from Ti vacancy or oxygen vacancy. In order to understand the origin of ferromagnetism, post-annealing of the nanorods was carried out at 773 K under Ar and oxygen atmospheres with the same gas flow rate to create oxygen-deficient and oxygen-rich environment, respectively. According to the SQUID measurement (Figure 7), there is not much difference on the magnetization in the undoped sample after annealing in different ambient. It is also noted that ferromagnetism is absent in the 0.5 at% Co-doped sample, and ferromagnetic ordering cannot be observed in 1 and 3 at% Co-doped samples.

**Figure 8:**
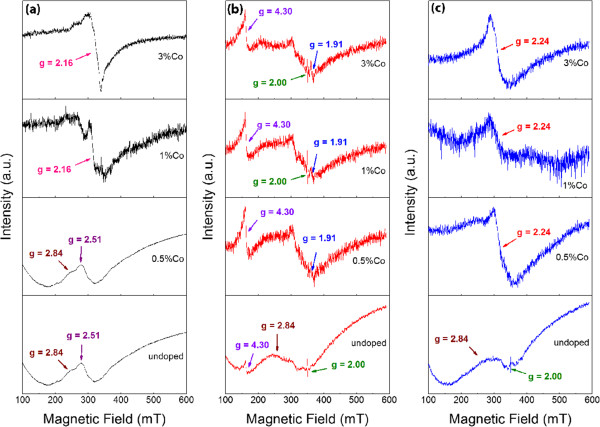
**ESR spectra of undoped and Co-doped TiO2 nanorods. (a)** As-prepared, **(b)** post-annealed in Ar atmosphere and **(c)** post-annealed in O_2_ atmosphere.

In order to understand the mechanism of ferromagnetism in the Co-doped TiO_2_ system, we performed ESR measurements, as shown in Figure 8. For the as-prepared samples, the undoped TiO_2_ and 0.5 at% Co-doped TiO_2_ have similar ESR spectra. The electron *g* factors are calculated from $g=\frac{h\nu }{\beta H_{O}}$, where *h* is the Planck constant (4.135 × 10^−15^ eV s), ν is the frequency (9.8 GHz), *β* is the Bohr magneton (5.788 × 10^−5^ eV·T^−1^) and *H*_o_ is the resonance magnetic field [43]. A strong resonance at around *g* = 2.84 and *g* = 2.51 is observed (Figure 8a). The ESR spectra in the whole magnetic field range are non-symmetric, which suggest ferromagnetic ordering [44,45]. For 1 and 3 at% Co-doped TiO_2_, strong paramagnetic resonance peak at *g* = 2.16 is observed, which is associated with the coupling of doped Co ions and oxygen vacancies [46]. This suggests that oxygen vacancy does not contribute to the ferromagnetic ordering in our samples. When the samples were performed post-annealing, the ESR spectra of all the samples change significantly (Figure 8b,c). For the undoped sample, the spectrum is still unsymmetrical and the resonance peak at *g* = 2.84 remains unchanged, while the resonance peak does not appear in the other three samples. The peak at *g* = 2.51 has also been reported by Guskos et al. [47]. However, there is no discussion or explanation on the origin of this peak. From Figure 8b, we can also see that free ions initiated from oxygen vacancy (*g* = 2.0) are detected. Such free ions may be attributed to the single electron trapped in an oxygen vacancy site [48,49]. In addition, we also observe Ti^3+^ in all the post-annealed samples due to the resonance peak at 1.91 [50]. The free ions of oxygen vacancy and Ti^3+^ observed in all the samples confirm that oxygen vacancy and Ti^3+^ are not the origin of ferromagnetism. It is noted that a strong resonance at *g* = 4.3 is detected for all the samples. It has been observed in commercial nanostructured TiO_2_ powders, which may be related to disordering or surface defects of nanostructured TiO_2_[51]. In Figure 8c, the peak related to surface defects (*g* = 4.3) can no longer be observed when the samples are annealed under oxygen atmosphere. The resonance at *g* = 2.24 is observed in the Co-doped samples, which is associated with Co ions combined with oxygen vacancies [46]. It has been known that an oxygen-rich environment is beneficial to the formation of Ti vacancy. However, the annealing conditions carried out in our work may not be able to create a sufficient amount of Ti vacancy due to its high formation energy. From Figure 8, it can be concluded that the ferromagnetic ordering is related to the resonance peak at *g* = 2.84, though the peak has not been reported before.

ESR results have suggested that Ti vacancies and correlated complex defects may be the origin of ferromagnetism. It is known that the formation energy of Ti vacancy is quite high [52]. For the undoped samples, Ti vacancies may be able to form due to the high calcination temperature. The sufficient oxygen partial pressure during synthesis may facilitate the creation of Ti vacancies. Co doping has strong influence of the synthesis process. The Co oxide coated on the surface of TiO_2_ nanoparticles may impede the formation of Ti vacancies. When the sample is performed with annealing under an oxygen-deficient environment, some amount of oxygen vacancies is formed, which may recombine with Ti vacancies. Hence, the ferromagnetism decreases.

## Conclusions

Rutile TiO_2_ nanorods have been successfully synthesized using molten salt method. Our results show that Co doping has strongly affected the morphology and phase transformation of TiO_2_ nanorods. A high doping concentration of Co (5 at%) results in the formation of nanoparticles rather than nanorods. By changing the growth conditions, the experimental results demonstrate that the calcination temperature is the dominant to control the sizes of nanorods not the amount of salt and heating duration.

Magnetic properties were investigated by SQUID and ESR measurements. As-prepared TiO_2_ nanorods without Co doping show weak ferromagnetism at room temperature. As the Co doping increased, the ferromagnetic ordering is weakened. The nanorods show paramagnetism or antiferromagnetism when the Co-doping concentration is beyond 1 at%. These results imply that the ferromagnetism may originate from the intrinsic point defects in the materials instead of Co doping. Detailed study by ESR indicates that the ferromagnetism is correlated to the Ti vacancy.

## Abbreviations

DMS: diluted magnetic semiconductor; DSP: dibasic sodium phosphate; ESR: electron spin resonance; ICPMS: inductively coupled plasma mass spectrometry; MBE: molecular beam epitaxy; PLD: pulsed laser deposition; SAED: selected area electron diffraction; SEM: scanning electron microscopy; SQUID: superconducting quantum interference device; TEM: transmission electron microscopy; XRD: X-ray diffractometery; XPS: X-ray photoelectron spectroscopy.

## Competing interests

The authors declare that they have no competing interests.

## Authors' contributions

LTT carried out the sample preparation, experimental measurements and drafted the manuscript. XL performed the ESR measurement. TTT carried out the SQUID measurement. SL participated in the manuscript revision. JBY conceived the work, supervised the experiments and revised the manuscript. All authors read and approved the final manuscript.
